# Assessment of thermal changes in water buffalo mobilized from the paddock and transported by short journeys

**DOI:** 10.3389/fvets.2023.1184577

**Published:** 2023-05-12

**Authors:** Daniela Rodríguez-González, Isabel Guerrero Legarreta, Rosy G. Cruz-Monterrosa, Fabio Napolitano, Cristiane Gonçalves Titto, Ayman H. Abd El-Aziz, Ismael Hernández-Avalos, Alejandro Casas-Alvarado, Adriana Domínguez-Oliva, Daniel Mota-Rojas

**Affiliations:** ^1^Master’s Program in Agricultural and Livestock Sciences [Maestría en Ciencias Agropecuarias], Universidad Autónoma Metropolitana (UAM), Xochimilco Campus, Mexico City, Mexico; ^2^Department of Biotechnology: Food Science, Universidad Autónoma Metropolitana, Iztapalapa Campus (UAM-I), Mexico City, Mexico; ^3^Department of Food Science, Universidad Autónoma Metropolitana (UAM-L), Campus Lerma, Lerma City, Mexico; ^4^Scuola di Scienze Agrarie, Forestali, Alimentari ed Ambientali, Università degli Studi della Basilicata, Potenza, Italy; ^5^Laboratório de Biometeorologia e Etologia, Faculdade de Zootecnia e Engenharia de Alimentos, FZEA-USP, Universidade de São Paulo, Pirassununga, Brazil; ^6^Animal Husbandry and Animal Wealth Development Department, Faculty of Veterinary Medicine, Damanhour University, Damanhour, Egypt; ^7^Department of Biological Science, FESC, Universidad Nacional Autónoma de México (UNAM), Cuautitlán Izcalli, Mexico; ^8^Neurophysiology, Behavior and Animal Welfare Assessment, DPAA, Universidad Autónoma Metropolitana (UAM), Xochimilco Campus, Mexico City, Mexico

**Keywords:** *Bubalus bubalis*, infrared thermography, animal welfare, transport, herding method

## Abstract

Evaluating the welfare of buffaloes during transport is key to obtaining and commercializing high-quality meat products; however, effective assessments require recognizing several stressors that activate physiological mechanisms that can have repercussions on the health and productive performance of species. The aim of this study was to evaluate the surface temperatures of different body and head regions in this species during events prior, and posterior, to transport for short periods; that is, from paddock to loading. The second goal was to determine the level of correlation between thermal windows. This study used infrared thermography (IRT) to evaluate the surface temperature of 624 water buffaloes (Buffalypso breed) during 12 short trips (average duration = 2 h ± 20 min) by focusing on 11 regions of the body (*Regio corporis*), in the head regions (*Regiones capitis*) the face regions (*Regiones faciei*), Orbital region (*Regio orbitalis*) with special attention to structures such as the lacrimal caruncle, periocular area and lower eyelid (*Regio palpebralis inferior*); nasal region (*Regio nasalis*) with special attention to nostril thermal window; and regions of the skull (*Regiones cranii*) such as auricular region (*Regio auricularis*) with special attention to auditory canal and frontal–parietal region (*Regio frontalis-parietalis*) and trunk region (*Truncus regionis*) such as thoracic and abdominal regions, regions of the vertebral column (*Columna vertebralis*) with the thoracic vertebral region (*Regio vertebralis thoracis*) and lumbar region (*Regio lumbalis*); and regions of the pelvis limb (*Regiones membri pelvini*). Recordings were made during seven phases: paddock (P1), herding (P2), corral (P3), chute handling (P4), shipping (P5), pre- (P6), and post-transport (P7). A total of 48,048 readings were obtained from 11 thermal windows. The results showed that the surface temperatures of the windows increased by as much as 5°C during P2, P3, P5, P6, and P7 compared to P1 and P4 (*p* < 0.0001). Differences of at least 1°C were also observed between thermal windows in the craniofacial, lateral corporal, and peripheral zones (*p* < 0.0001). Finally, a strong positive correlation (*r* = 0.9, *p* < 0.0001) was found between the thermal windows. These findings lead to the conclusion that the surface temperature of the craniofacial and corporal regions of buffaloes transported for short periods varied in relation to the phase of mobilization (from paddock to post-transport), likely as a response to stressful factors, since herding and loading increased the thermal values in each window. The second conclusion is that there are strong positive correlations between central and peripheral thermal windows.

## 1. Introduction

Animal transport is an integral element in the process of breeding and fattening buffaloes (1–3) to obtain and commercialize meat products (4–7). However, transport is considered one of the most stressful procedures for farm animals because it exposes them to environmental factors, drivers’ aptitude, road conditions, and trip duration, all of which can affect their welfare by provoking injuries or negative mental states such as fear and anxiety, due to the triggering of physiological and behavioral mechanisms that can modify muscular metabolism and muscle-to-meat conversion, and affect the amount, quality, and innocuity of the final products (8–11). Obviously, this means economic losses for the producers.

In light of the negative effects of mobilization-induced stress, diverse behavioral, pathological, and physiological indicators are being used to evaluate the levels of animal welfare during this phase, where observable physiological changes include dehydration, hyporexia, tissue damage, smoke inhalation, and physical, thermal, and psychological stress (12–15), which can inhibit the immune system under long exposure to adverse environments (16). Endocrine and metabolic alterations, increased plasma cortisol and serum protein concentrations, and glycemia levels have been documented after 16 h of transport, while loss of live weight (from 7.9 to 10.5%) (17, 18) has been associated with energy deficits caused by muscular and hepatic glycogenolysis (19). Another physiological indicator suggested for monitoring is body temperature, as increases can aid in recognizing stressful processes and may be related to the overall health condition of animals (20). Regarding behavioral changes in buffaloes, reports have emphasized that transport conditions increase the frequency of aggressive and dominant behaviors (21, 22), urination (28%), defecation, and urination and defecation (72%), as well as attempts to jump from vehicles (12%) (7) which results in falls and injuries that affect both the animals and the quality of the final product.

Water buffaloes have shown a greater predisposition to transport-induced stress due to certain morphophysiological characteristics that make them susceptible to thermal stress, a very common consequence of transport. Buffaloes have fewer sweat glands and a lower proportion in relation to body weight than other livestock species (394/cm^2^), low hair density (100–200/cm^2^), and hide with more pigmentation that impedes adequate thermal exchange (23, 24). Evaluating body temperature in buffaloes is a parameter that permits the determination of the degree of thermal stress. The “gold standard” for monitoring this factor was obtained via invasive methods, like measuring rectal and vaginal temperatures (25). However, these techniques can generate stress in animals due to the handling requirements. For this reason, non-invasive technologies such as IRT have been proposed as tools to evaluate surface temperatures in these animals (26, 27).

IRT enables evaluation of the amount of heat that a body radiates, a phenomenon related to changes in peripheral blood flow through activation of the autonomic nervous system (ANS) in the face of diverse stressors (28). Because transport entails a certain degree of stress that has not been widely studied in water buffaloes, the aim of this study was to evaluate the surface temperatures of different body and craniofacial regions in this species during events prior, and posterior, to transport for short periods; that is, from paddock to loading. The second goal was to determine the level of correlation between central (abdominal and thoracic region) and peripheral (limb and nasal) thermal windows.

## 2. Materials and methods

### 2.1. Study location

The study was conducted in the state of Veracruz in south-southeast Mexico, from June 2021 to August 2022. The production unit is in a zone with a tropical humid climate, with a mean temperature of 31 ± 2°C, relative humidity of 86%, an elevation of 20 m.a.s.l., and annual rainfall of 1,500–2,000 mm (29).

### 2.2. The animals and the distribution of the phases

For this study, 624 male Buffalypso buffaloes destined for fattening were selected. The mean weight of the animals was 230 ± 21.78 kg. They were transported on 12 short trips covering 110 km with a mean duration of 2 h ± 20 min and an average velocity of 55 km/h.

The buffaloes were divided for the 12 trips according to the capacity of the first floor of the truck as follows: 53, 51, 49, 56, 53, 50, 53, 52, 54, 51, 52, and 50. Before each trip, they were housed in the same paddock and herded gently (no use of physical utensils or shouting by handlers).

### 2.3. Housing prior to loading and vehicle type

**Figure 1:**
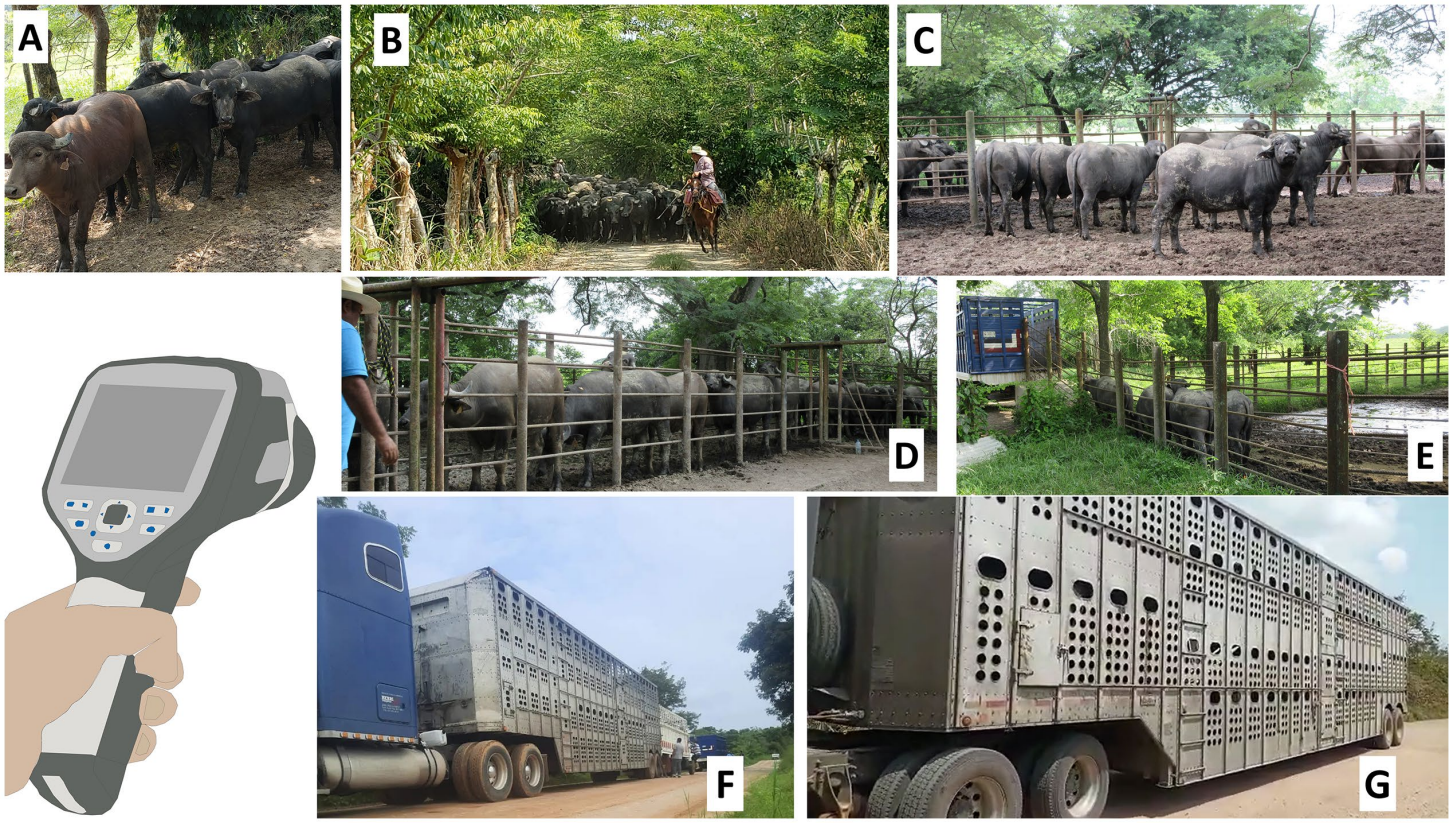
Areas where the evaluation phases were carried out. **(A)** The paddock of the production unit; basal sampling (P1). **(B)** After herding by men on horseback (P2). **(C)** After spending the night in the cattle corral system, prior to entering the handling chute (P3). **(D)** Handling chute (P4). **(E)** Entering the vehicle via a loading ramp (P5). **(F,G)** The vehicle prior to transport (P6) and upon arrival at the reception site (P7).

To carry out the experimental protocol, the transport process was divided into seven phases (Figure 1):

Phase 1 (P1). Paddock, basal monitoring: The animals were housed in paddocks with natural shade until they reached a weight of 208–251 kg. Alimentation consisted of native grasses (*Paspalum fasciculatum*) (percentage of dry matter = 14.7% with 56.7% neutral detergent fiber, 38.8% acid detergent fiber, 6.2% raw protein (30), and 50 g/animal of mineral complements).
Phase 2 (P2). Herding by handlers on horseback: Once the steers reached the desired weight, they were herded gently by men on the horseback without physical utensils or electric prods. For all trips, herding from the paddock to the corral system (P3) required an average of 25–35 min. This was always performed at 18:00. The animals remained in the cattle corral system for 12 h (including the night before transport).
Phase 3 (P3). Cattle corral system: The pen had a surface area of 630 m^2^ and a dirt floor. It was framed by 1.6 m high tubular structures with an entrance gate and an exit gate that led to the handling chute. The buffaloes remained there for 8 h without solid food but with *ad libitum* access to water.
Phase 4 (P4): Handling chute: The chute consisted of a hallway (1 m wide × 1.6 m high × 3.5 m long), framed by tubular structures, and connected to an individual weight scale. This hallway led to a loading ramp. The average duration was 50 min.
Phase 5 (P5). Loading: During an interval of approximately 30–50 min, the buffaloes were led up with a concrete loading ramp made of anti-slip grooving. Slope was 20°.
Phase 6 (P6). Pre-transport: The time spent in the vehicle before transport, counted from the moment when all the animals had been loaded into the truck.
Phase 7 (P7). Post-transport: The time when the animals were still inside the vehicle, immediately upon arrival at the reception site, after transport over dirt and paved roads.

The topography of the roads was classified as unpaved or paved. The former had the specifications of Class E roads, according to the classification of Mexico’s Department of State (*Secretaría de Gobernación*) (31), with a maximum slope of 13%.

The mean maximum velocity was 25 km/h. Dirt roads were used to exit the production unit (10 km) and enter the reception site (12.5 km). The total trip length on the unpaved roads was 22.5 km with a maximum overelevation of 10%, vertical curves of 4 m/%, and road width of 6 m. The other 87.5 km were covered on a paved road with a maximum transversal slope of 7%, a maximum speed of 55 km/h, vertical curves of 5 m/%, and a road width of 8 m.

The vehicle was a Wilson Trailers Silver Star (dimensions: 15.24 m long × 2.59 m wide × 4.6 m high) with a load capacity of 22,500 kg. The truck was designed especially for animal transport with two floors, sliding side doors, a rear guillotine door, a non-slip floor, a reinforced plastic roof, and fiberglass. The walls were made of galvanized steel and aluminum, with openings for ventilation during the trips.

### 2.4. Monitoring by infrared thermography

**Figure 2:**
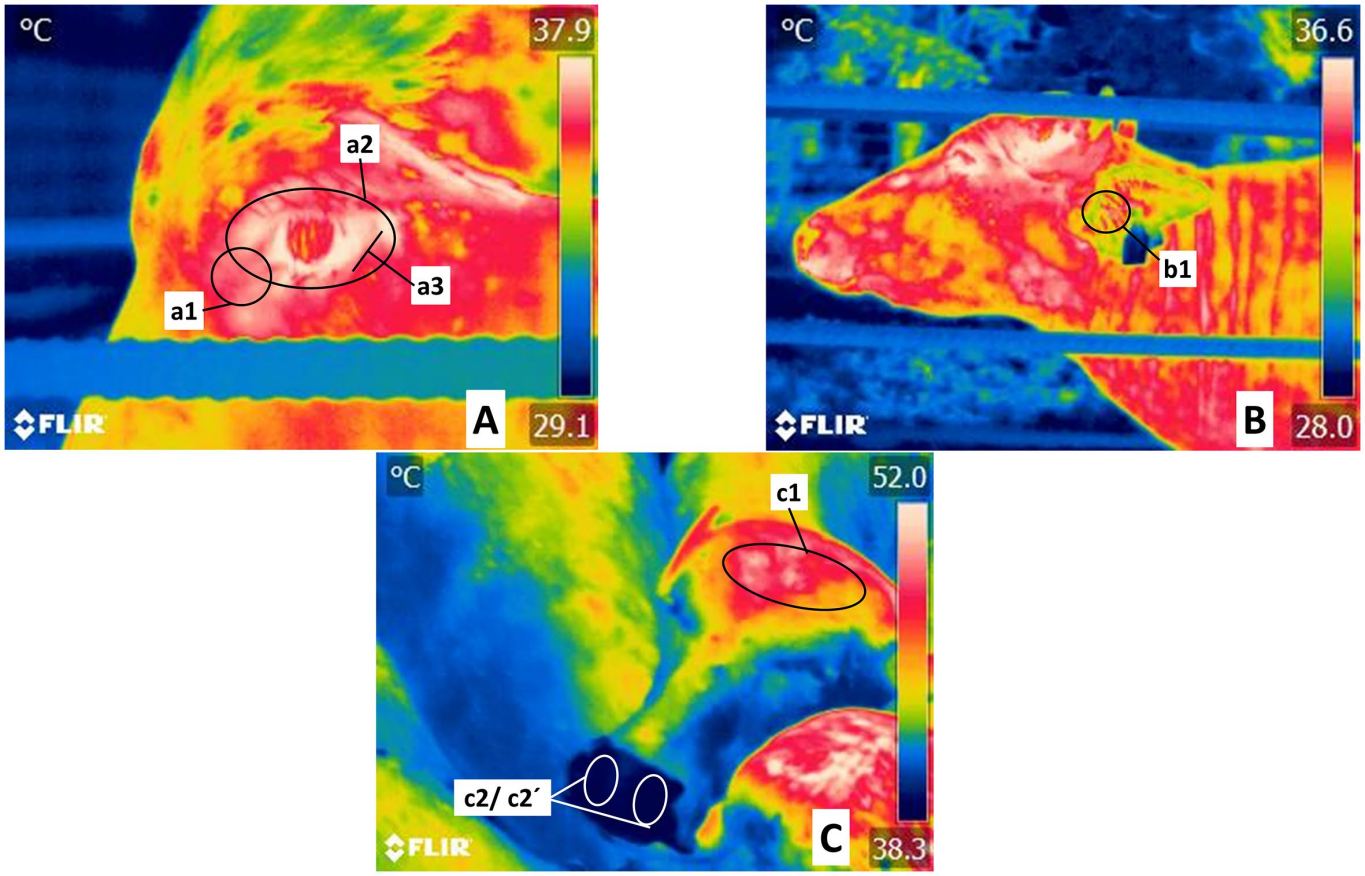
Delimitation of thermal windows in the head regions (*Regiones capitis*). **(A)** Ocular thermal windows in Orbital region (*Regio orbitalis*). Recordings in this zone centered on the lacrimal caruncle, delimited as circle a1, and considered the medial commissure of the eyelids up to half a centimeter toward the cranium. This area reflects the radiation of blood flow from the infraorbital artery innervated by the sympathetic branches of the facial nerve. The periocular thermal window, marked by circle a2, spans the periphery of the upper and lower eyelids, irrigated by both the supraorbital and infraorbital arteries. The lower eyelid thermal window, defined by a line approximately 3 cm long (a3), was used to evaluate radiation from the conjunctival mucosa and was irrigated by the infraorbital artery. **(B)** Regions of the skull (*Regiones cranii*), thermal window of the auricular pavilion in the auricular region (*Regio auricularis*). This area is framed by circle b1 in the region of the auditory canal, the antihelix ridge, and the lateral peduncle of the helix, irrigated by the media and the lateral and central auricular arteries. **(C)** Thermal window of the frontal-parietal region (*Regio frontalis-parietalis*). Outlined by an oval (c1) that encompasses the frontal-parietal region (*Regio frontalis*), cornual region (*Regio cornualis*) limits, and parietal region (*Regio parietalis*), a zone irrigated by cornual and supraorbital arteries. *Regio naris* thermal window in the frontal region (*Regio frontalis*): ovals c2 and c2´. It was used to evaluate the radiation from the maxillary artery.

Thermographic monitoring was performed using a FLIR^®^ Thermal TM E60 infrared camera (FLIR Systems, United States) with an IR resolution of 320 × 240 pixels, thermal sensitivity <0.045°C, and precision ±2°C or 2%. All the radiometric images were taken at a distance of 1–1.5 m from the buffaloes, focusing on one of the following regions: facial regions (*Regiones faciei*) and skull region (*Regiones cranii*), trunk region (*Truncus regionis*) left or right, or regions of the vertebral column (*Columna vertebralis*), and regions of the pelvis limb (*Regiones membri pelvini*). Eleven body thermal windows in the head (*Regiones capitis*) and the trunk (*Truncus regionis*) were selected and delimited using FLIR tools software. The facial regions (*Regiones faciei*) included the orbital region (*Regio orbitalis*) with special attention to structures such as the lacrimal caruncle, periocular area and lower eyelid (*Regio palpebralis inferior*); nasal region (*Regio nasalis*) with special attention to nostril thermal window and regions of the skull (*Regiones cranii*) such as auricular region (*Regio auricularis*) with special attention to auditory canal and frontal–parietal region (*Regio frontalis-parietalis*) (Figure 2).

**Figure 3:**
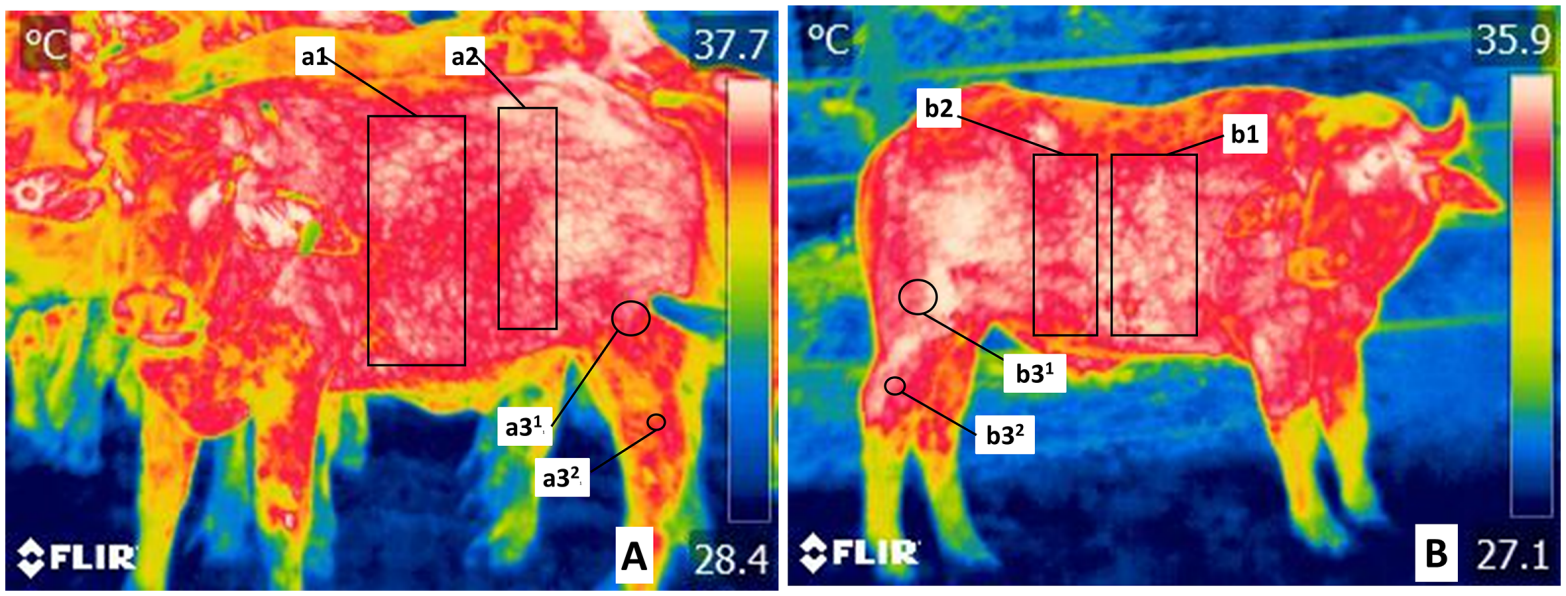
Delimitation of the trunk thermal windows in the trunk region (*Truncus regionis*). **(A)** The thermal window of the thoracic region in costal region (*Regio costalis*) is shown by a rectangle (a1, b1) traced from the costal arch from 1° to 12°. The cranial abdominal region (*Regio abdominis cranialis*) delimited by a rectangle (a2, b2), includes the oblique abdominal and rectus abdominis muscles irrigated by the surface epigastric artery (*A. epigastrica cranialis superficiales*). **(B)** The thermal window of the regions of the pelvis limb (*Regiones membri pelvini*) were marked by two circles at the *Regio femoris* (a3^1^, b3^1^), and at the *Regio tarsi* (a3^2^, b3^2^). It includes the femoral region the femoral muscle and the tarsus region lateral projection of the region of the common calcaneal tendon (*Regio tendinis calcanei communis*), regions irrigated by the femoral artery, and branches of the saphenous artery.

The trunk region (*Truncus regionis*) thoracic region and abdominal region, thermal window of the trunk, and regions of the pelvis limb (*Regiones membri pelvini*) with two anatomical regions indicated the femoral region (*Region femoris*) and the tarsus region (*Regio tarsi*), thermal windows of the regions of the pelvis limb (*Regiones membri pelvini*) are shown in Figure 3.

**Figure 4:**
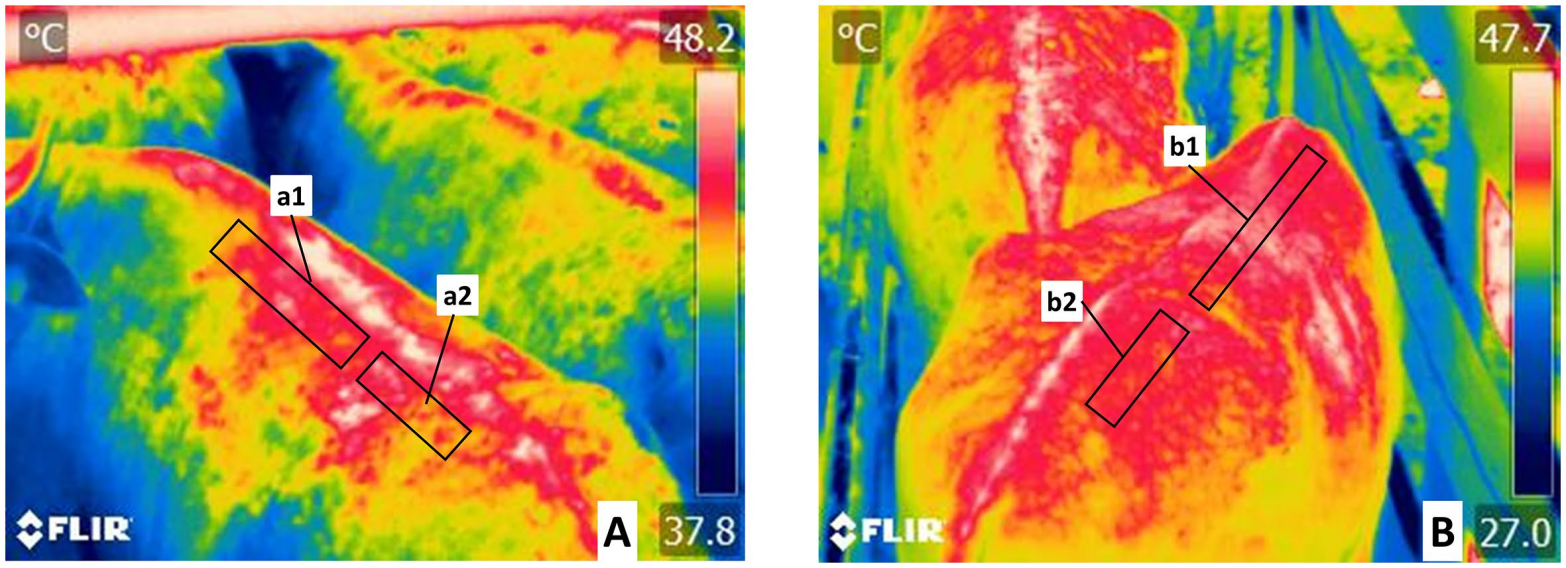
Delimitation of the regions of the vertebral column (*Columna vertebralis*) thermal windows. **(A)** The thermal window of the thoracic vertebral region (*Regio vertebralis thoracis*) is marked with a rectangle. It includes the left (a1) and right (b1) projections from the thoracic vertebrae and the thoracic vertebrae and the with of the transverse processes from thoracic vertebrae 1ª–12ª. This area is irrigated by spinal branch (*Ramus spinalis*) from the dorsal branches of the abdominal aorta artery, in the thoracic vertebral region. **(B)** The thermal window of the lumbar vertebral region (*Regio vertebralis lumbalis*). This rectangle encompasses the left (a2) and right (b2) projections from the lumbar vertebrae above the transverse processes of lumbar vertebrae 1ª–7ª. This permits the evaluation of radiation from the spinal branch (*Ramus spinalis*) from the dorsal branches of the abdominal aorta artery, in the lumbar vertebral region.

Figure 4 shows the regions of the vertebral column (*Columna vertebralis*) with the thoracic vertebral region (*Regio vertebralis thoracis*) and lumbar region (*Regio lumbalis*).

### 2.5. Recording and reading of the IRT

In accordance with the seven experimental phases, IRT readings were recorded as follows: P1, prior to the buffaloes being herded by men on horseback. Recordings were made while they were at rest for 1 h under shade, between 17:00 and 18:00 h; P2, inside the corral on the day prior to transport; P3, at 08:00 h, prior to guiding the animals into the handling chute to be weighed and loaded; P4, while the buffaloes were in the handling chute waiting to be weighed; P5, during loading as the animals walked up the ramp and entered the truck; P6, for 40 min after all the buffaloes were loaded into the truck but with the motor shut off, and P7, for 40 min immediately after concluding the short trip, but with the truck’s motor still turned on.

All digital and radiometric images were stored in JPG format for later analysis using FLIR software (Tools Systems, United States) to obtain the maximum, minimum, and average temperature readings, which were then entered into a database.

The ambient temperature (AT) and relative humidity (RH) were monitored at different times, in accordance with the experimental phase. P1: AT and RH ranged from 23 to 25°C and 81–90%, respectively (17:00–18:00 h). P2: AT and RH values were 21–25°C and 81–88%, respectively (18:00–18:30 h). P3: AT and RH fluctuated around 20–23°C and 81–86%, respectively (08:00 h). P4: AT and RH were 22–25°C and 81–88%, respectively (09:00 h). P5: values were 22–27°C and 81–88% at 10:00 h. P6 and P7: data for AT and RH ranged from 22–28°C and 81–89% at 11:00 h and 23–30°C and 83–94% at 14:00–15:00 h.

### 2.6. Statistical analysis

The GraphPad Prism statistical package (ver. 9.4.1) was used for all analyses. First, descriptive statistics were obtained for each thermal window (periocular, lacrimal caruncle, lower eyelid, auditory canal, nostrils, parieto-frontal area, and the thoracic, abdominal, appendicular, lumbar, and dorsal areas) and event (P1–P7). Results are expressed as mean ± standard error (SE). The Shapiro-Wilk test was used for normality analysis.

The events and thermal windows were considered as independent variables, and the surface temperature was considered as the dependent variable. Analysis of variance (ANOVA) was performed to evaluate the effect of these variables in a linear mixed model using the following statistical model:


$$Y_{ijak}=\mu +\tau i+\tau j+\tau i\tau j+\beta k+e_{ijk}$$

where:

*Y* = response variable (surface temperature).

*τi* = effect of the thermal window.

*τj* = effect of the event.

*τiτj* = interaction effect.

*β* = random effect (animal).

*μ* = population mean.

*e* = error.

Differences between means were analyzed using a *post-hoc* Tukey test. The level of significance was set at *p* < 0.05. Correlation analysis was conducted using the Pearson’s correlation coeficient.

### 2.7. Ethics statement

The experimental protocol was approved by the Scientific Commission of the Master in Science (CAMCA) “Maestría en Ciencias Agropecuarias” of the Faculty of Veterinary Medicine and Animal Husbandry, Universidad Autónoma Metropolitana, Mexico City, Mexico. The animals used in this study were handled gently, without the use of physical utensils that could cause stress, in accordance with the guidelines of the Official Mexican Standards NOM-062-ZOO-1999, which establish technical specifications for the production, care, and ethical use of animals during experimentation; and NOM-051-ZOO which establish technical specifications for humanitarian handling in the mobilization of animals published by the Department of Agriculture, Rural Development, Fisheries and Alimentation. It is also important to consider that the animals were not touched or stressed, since infrared thermography is a non-invasive technique.

## 3. Results

During the seven phases analyzed, we obtained 48,048 surface temperature readings from 11 thermal windows of 624 water buffaloes transported on 12 short trips. Table 1 shows the mean temperatures of the thermal windows in the head regions (*Regiones capitis*) and their corresponding standard errors.

**Table 1:** Mean ± standard error (SE) temperatures for the thermal windows of the head regions (*Regiones capitis*) of 624 water buffaloes in the 7 phases of transport for short periods.

					Phase			
Thermal window		P1. Paddock	P2. Herding	P3. Corral	P4. Handling chute	P5. Loading	P6. Pre-transport	P7. Post-transport
**Facial region** (*Regions faciei*)	**Orbital region** *(Regio orbitalis)* Lacrimal caruncle	33.9 ± 0.03^e,1^	38.9 ± 0.06^c,2^	37.4 ± 0.02^e,2^	38.7 ± 0.03^c,3^	38.3 ± 0.03^d,3^	40.2 ± 0.04^b,2^	41.1 ± 0.09^a,4^
	Periocular region	33.7 ± 0.03^f,2^	39.0 ± 0.04^c,2^	37.7 ± 0.02^e,1^	39.0 ± 0.03^c,2^	38.5 ± 0.03^d,2^	40.0 ± 0.03^b,2,3^	41.5 ± 0.09^a,2^
	Lower eyelid	33.9 ± 0.04^f,1,2^	38.3 ± 0.02^c,3^	36.4 ± 0.06^e,3^	38.1 ± 0.04^c,4^	37.7 ± 0.02^d,4^	40.3 ± 0.08^b,2^	41.3 ± 0.09^a,3^
	**Nasal region** (*Regio nasalis*) Nostrils	31.7 ± 0.04^g,3^	36.4 ± 0.04^c,4^	35.1 ± 0.03^f,5^	35.6 ± 0.02^e,6^	35.9 ± 0.03^d,6^	37.3 ± 0.03^b,4^	38.5 ± 0.03^a,6^
**Regions of the skull** (*Cranii regions*)	Auricular pavilion	31.2 ± 0.05^f,3^	38.9 ± 0.03^b,2^	36.3 ± 0.05^e,4^	37.3 ± 0.03^c,5^	37.1 ± 0.02^d,5^	39.8 ± 0.03^a,3^	39.7 ± 0.04^a,5^
	Frontal-parietal region	31.5 ± 0.06^g,3^	43.1 ± 0.08^c,1^	36.7 ± 0.03^f,3^	45.1 ± 0.07^b,4^	41.2 ± 0.06^e,1^	41.1 ± 0.09^e,1^	49.7 ± 0.15^a,1^

Different literals a, b, c, d, e, f, g indicate statistically significant differences among the temperatures of the animals according to the phase.

Different numbers 1, 2, 3, 4, 5, 6 indicate statistically significant differences between the temperatures of the thermal windows.

Observations showed the thermal windows considered in the orbital region (*Regio orbitalis*) such as the lacrimal caruncle, periocular region, and lower eyelid thermal windows had significant average increases of 4.3°C, 4.8°C, and 3.7°C, respectively, during P2, P3, and P4, compared to P1 (*p* < 0.0001). Readings for the Regions of the skull (*Cranii regions*), the auditory canal thermal window showed increases of 1.3°C, 0.8°C, and 1.3°C in those windows compared to P5 (*p* < 0.0001), while the comparison of P3 to P2 and P4 revealed a significant reduction of 1.3° (*p* < 0.0001). Observations during P6 and P7 showed a gradual increase in surface temperature compared with P5 (*p* < 0.0001).

Readings for the auditory canal window showed an increase of 7.7°C from P1 to P2 (*p* < 0.0001), but the auricular temperature decreased by 2.6°C from P2 to P3 (*p* < 0.0001). Later, it increased by 1°C and 0.8°C during P4 and P5, respectively, compared to P2 (*p* < 0.0001). Recordings for P6 and P7 showed significant increases of 2.7°C and 2.5°C compared to P4 and P5 (*p* < 0.0001).

For the thermal windows the nasal region (*Regio nasalis*), the nostrils and frontal-parietal region, the study found that temperatures in P1 were 4.7°C and 11.6°C lower, respectively, than those in P2 (*p* < 0.0001), but those temperatures decreased by 1.3°C and 6.4°C, respectively, in P3 (*p* < 0.0001). In P4 and P5, the temperatures increased by 0.5°C and 0.8°C, respectively (*p* < 0.0001). This increase was maintained in P6 (2°C) and P7 (2.6°C), compared to P5 (*p* < 0.0001). Readings from the thermal window of frontal- parietal region showed increases of 8.4°C in P4, 4.5°C in P5 (*p* < 0.0001), and 8.6°C in P7 compared to P5 and P6 (*p* < 0.0001).

Considering the different phases of this study and the thermal responses in the windows described, during P1, temperatures in the orbital region (*Regio orbitalis*) such as the lacrimal caruncle, periocular, and lower eyelid were significantly 2°C higher than in the auditory canal, nostrils, and thermal window of frontal-parietal region (*p* < 0.0001). During P2, the parieto-frontal window presented the highest temperature among all windows (*p* < 0.0001). The orbital region (*Regio orbitalis*) such as the lacrimal caruncle, periocular, and auditory canal had temperatures 0.7°C higher than those of the lower eyelid (*p* < 0.0001), but the latter was 1.9°C higher than the nostrils window (*p* < 0.0001).

Observations from P3 showed that the temperature of the periocular window was 0.3°C higher than that of the lacrimal caruncle (*p* < 0.0001), and 1°C higher than that of the lower eyelid (*p* < 0.0001). Similarly, the temperature of the lower eyelid was 1.2°C higher than that in the nostril window (*p* < 0.0001). For P4, P5, P6, and P7, the thermal window of frontal–parietal region showed the highest temperatures in all regions (*p* < 0.0001), followed in the orbital region (*Regio orbitalis*) by the lacrimal caruncle, periocular region, and lower eyelid region (*p* < 0.0001).

The temperatures of the thermal windows of the lateral region of the trunk are presented in Table 2. In P1, the thermal window of the thoracic region presented a lower temperature than the other phases but was 8.6°C lower in P2 (*p* < 0.0001). Readings for phases P3, P4, and P5 showed reductions of 5.4°C, 3°C, and 3.3°C, respectively, compared to P2 (*p* < 0.0001), but increases of 3.4°C in P6 and 4.9°C in P7 were determined (*p* < 0.0001). The thermal window of the abdominal region recorded a temperature increase of 6.8°C in P2 compared to P1 (*p* < 0.0001). With respect to P3, P4, and P5, we observed decreases of 4°C, 1.7°C, and 2.8°C, respectively, compared to P2 (*p* < 0.0001), whereas P6 and P7 registered increases of 4.6°C and 5.3°C, respectively (*p* < 0.0001). Temperature readings for the thermal window of the pelvic limb in P1 were the lowest of all events (*p* < 0.0001), but the temperatures recorded in P2 and P6 were significantly higher than those in P3, P4, and P5 by 2.7, 0.2, and 2.3°C, respectively (*p* < 0.0001).

**Table 2:** Mean ± standard error (SE) of the temperatures in the thermal windows of the lateral region of the trunk of 624 water buffaloes transported during seven phases of short trips.

					Phase			
Thermal window		P1. Paddock	P2. Herding	P3. Corral	P4. Handling chute	P5. Loading	P6. Pre-transport	P7. Post-transport
Lateral region of the trunk	Thoracic region	33.0 ± 0.03^e,2^	41.6 ± 0.06^b,1^	36.2 ± 0.02^d,1^	38.6 ± 0.03^c,1^	38.3 ± 0.07^c,1^	41.7 ± 0.06^b,2^	43.2 ± 0.08^a,1^
	Abdominal region	33.5 ± 0.02^g,1^	40.3 ± 0.05^c,2^	36.3 ± 0.03^f,1^	38.6 ± 0.03^d,1^	37.5 ± 0.05^e,2^	42.1 ± 0.07^b,1^	42.8 ± 0.08^a,2^
Limbs region	Pelvic limb	32.1 ± 0.04^f,3^	38.7 ± 0.02^b,3^	35.9 ± 0.03^e,2^	38.4 ± 0.03^c,1^	36.3 ± 0.02^d,3^	38.6 ± 0.04^b,3^	39.1 ± 0.04^a,3^

Different literals a, b, c, d, e, f, g indicate statistically significant differences among the temperatures of the animals according to the phase.

Different numbers 1, 2, 3 indicate statistically significant differences between the windows.

For P1 and P6, we found that the thermal window had values 0.5 and 0.4°C above those of the thermal window of the thoracic region (*p* < 0.0001). Readings for the latter, compared to the limbs regions, showed increases of 0.9 and 3.7°C, respectively (*p* < 0.0001). During P2 and P7, the study found that the thermal window of the thoracic region was 1.3 and 0.4°C higher than the thermal window of the abdominal region (*p* < 0.0001) while the latter was 1.6 and 3.7°C higher than the thermal window of the pelvic limb regions (*p* < 0.0001).

Table 3 presents the thermal values recorded in the thermal windows of the regions of the vertebral column. The reading for the thermal window of the thoracic vertebral region (*Regio vertebralis thoracis*) in P1 was lower than in all other events (*p* < 0.0001). The temperature of this window was 6°C and 6.6°C higher in P2 and P4 than in P3 (*p* < 0.0001) but decreased by 2.1°C and 1.9°C in P5 and P6, respectively (*p* < 0.0001). Later, the surface temperature of this thermal window increased by 4.3°C compared to P6 (*p* < 0.0001). Observations of the thermal window of the lumbar region (*Regio lumbalis*) showed that P6 had the highest temperature of all thermal windows (*p* < 0.0001), although in P1, it obtained the lowest value (*p* < 0.0001). In P2 and P4, this zone registered temperatures of 6.4°C and 6.7°C, respectively, higher than in P3 (*p* < 0.0001). Finally, the surface temperature decreased in P5 and P6 by 1.9°C and 1°C, respectively, compared to P4 (*p* < 0.0001).

**Table 3:** Mean ± standard error (SE) temperatures for the thermal windows of the trunk region (*Truncus regionis*) left or right of 624 water buffaloes during 7 phases of short periods of transport.

	Phase						
Thermal window	P1. Paddock	P2. Herding	P3. Corral	P4. Handling chute	P5. Loading	P6. Pre-transport	P7. Post-transport
Thoracic vertebral region	32.1 ± 0.05^f,2^	41.6 ± 0.09^b,1^	35.6 ± 0.04^e,1^	42.2 ± 0.06^b,1^	40.1 ± 0.10^d,1^	40.8 ± 0.08^c,2^	45.1 ± 0.11^a,2^
Lumbar vertebral region	33.4 ± 0.04^g,1^	42.0 ± 0.08^c,1^	35.6 ± 0.04^f,1^	42.3 ± 0.03^b,1^	40.4 ± 0.13^e,1^	41.4 ± 0.07^d,1^	44.6 ± 0.07^a,1^

Different literals a, b, c, d, e, f, g indicate statistically significant differences between the temperatures of the animals according to the phase.

Different numbers 1, 2 indicate statistically significant differences between the thermal windows.

Regarding statistically significant differences between the thermal windows of the thoracic vertebral region and lumbar region, the findings showed that in P1 and P6, the temperatures of the thermal window of the lumbar region were 1.3 and 0.6°C higher, respectively, than in the thermal window of the thoracic vertebral region (*p* < 0.0001). At P7, the temperature of the thermal window of the thoracic vertebral region was 0.5°C higher than that in the thermal window of the lumbar region (*p* < 0.0001).

Table 4 shows the level of correlation observed between the 11 thermal windows. Overall, the correlations among the different thermal windows were positive, strong, and statistically significant (*p* < 0.0001).

**Table 4:** Correlations between the thermal windows of 624 water buffaloes during all seven phases of short periods of transport.

	Lacrimal caruncle	Periocular region	Lower eyelid	Auricular pavilion	Nostrils	Frontal-parietal region	Thoracic region	Abdominal region	Limbs region	Lumbar region	Thoracic vertebral region
Lacrimal caruncle	1*	0.96*	0.97*	0.94*	0.95*	0.95*	0.94*	0.94*	0.95*	0.95*	0.93*
Periocular region		1*	0.97*	0.99*	0.99*	0.98*	0.97*	0.96*	0.99*	0.97*	0.95*
Lower eyelid			1*	0.96*	0.97*	0.98*	0.97*	0.97*	0.96*	0.98*	0.97*
Auricular pavilion				1*	0.99*	0.96*	0.96*	0.94*	1*	0.96*	0.95*
Nostrils					1*	0.98*	0.98*	0.97*	0.99*	0.98*	0.97*
Frontal-parietal region						1*	1*	1*	0.96*	0.99*	0.99*
Thoracic region							1*	1*	0.96*	0.99	0.99*
Abdominal region								1*	0.95*	0.99*	0.98*
Limbs region									1*	0.97*	0.96*
Lumbar region										1*	0.99*
Thoracic vertebral region											1*

*p < 0.0001; ^NS^*p* > 0.05.

## 4. Discussion

The results obtained for the buffaloes transported for short periods showed that the surface temperatures of thermal windows of the head and trunk increased significantly in P2, P4, P6, and P7, that is, during the events that required handling (*p* < 0.0001). These responses may be associated with the loss of thermal stability in the buffaloes, a species whose optimal ambient for achieving thermoneutrality is in the range of 13–18°C (32, 33).

Although adequate temperatures have been reported for water buffaloes, there is limited literature on the thermal responses of these species during transport. For conventional bovines, a study on the transport by land and sea of 481 animals reported a significant association between increases in such biomarkers as glucose, creatine kinase, and lactate levels with IRT temperature readings (34). A study of 120 pigs transported for 40 min reported temperature increases in the orbital region and behind the ear, which correlated positively with increased salivary cortisol concentrations (*r* = 0.49 and *r* = 0.50, respectively) (35). Studies of this type in other livestock species subjected to transport suggest that surface temperatures can be useful indicators of physiological changes triggered in response to transport-induced stress (36, 37), which may be reflected as central and peripheral nervous system reply with activation of vasomotor modulation hyperthermia (38).

In our study, the largest surface temperature increases in the buffaloes were recorded in the post-transport phase (*p* < 0.0001). This can be explained by the activation of Autonomic nervous system (ANS) and its sympathetic branch, which generates catecholamine production as a short-term response (24). Catecholamines act on metabolically active organs such as brown adipose tissue to initiate thermogenesis, thus increasing heat irradiation (39, 40). Activation of the hypothalamic-pituitary-adrenal axis (HPA) causes the secretion of glucocorticoids (e.g., cortisol) that promote gluconeogenesis and lipolysis (41, 42). In this regard, studies on ruminants have reported increases in plasma cortisol by up to 10-fold after 30 and 45 min, 1 h, 2 h, and up to 4 h post-transport (43–47). Mitchell et al. (48), cited in Ali-Gholi and Daryoush (49), stated that sympathetic, adrenal, and medullar reactions are related to physiological responses during transport, while hypothalamus, hypophysis, and suprarenal reactions are responsible for cortisol secretion under adverse environmental conditions. Therefore, this physiological response is responsible for heat production during the perception of stressful factors under conditions of transport, handling, and environmental challenges.

This explanation may aid in understanding the report by Sakakibara et al. (50), who found a weak negative correlation between blood cortisol concentrations and surface temperatures (*r* = −0.209) in five bovines transported for 8 h. Robertson et al. (51) observed that activation of the ANS leads to catecholamine secretion (52) that can generate physiological effects like tachycardia, tachypnea, and hyperthermia (53, 54). This could be related to the level of stress experienced by the animals, as Hagenmaier et al. observed (55) in 80 calves transported under two levels of handling. Animals under intensive handling, with the use of electric prods and minimal trotting during loading, had higher lactate, epinephrine, norepinephrine, cortisol, and glucose levels but lower levels of blood pH and bicarbonate, with excess bases, compared to the animals that received minimal handling during transport. This means that the external factors which increase the stress response of animals during transport (e.g., higher surface temperatures) include social interaction with handlers and the training that stockpeople receive to mobilize animals of different species, two factors that are fundamental for preventing intense stress responses, even during short trips like those in the present study (2 h ± 20 min). Some authors have reported that even when the trip time is maintained below 12 h, some species are unable to become habituated immediately to the intrinsic and individual factors that cause stress (56, 57).

Another important condition observed during animal transport that needs to be emphasized is neophobia, that is, fear of new situations. This may be another factor involved in stress responses prior to transporting water buffaloes (58, 59). This fear involves activation of the basolateral region of the amygdala, which coordinates responses by the cerebral cortex and hypothalamus, the physiological center that modulates heart and respiratory rates, and body temperature during events that cause fear or anxiety (60, 61).

With respect to the temperature differences detected among the different thermal windows, the minimal increase of 1°C among the facial, skull and nasal regions in comparison with lateral corporal trunk limbs region, and lateral region of the trunk (*p* < 0.0001) can be explained by the extensive distribution of capillaries and arteriovenous anastomoses that allow heat exchange with the environment (62). When mechanisms that preserve thermoneutrality are activated, heat loss by evaporation (as water vapor), convection, or conduction is responsible for the changes in the amount of heat irradiated through the skin (38, 63). Specifically, the thermal windows of head and periocular, and frontal-parietal regions showed a difference of 4°C (*p* < 0.0001), possibly due to distribution of the blood vessels in those areas. For example, the head regions is were irrigated by the facial artery and its branches in the infraorbital artery, which carries blood to the lower eyelid and lacrimal caruncle (24, 64), whereas the frontal-parietal region is supplied by branches (*A. transversa faciei*, *A. auricularis rostralis*, *A. palpebralis inferior lateralis*, *A. palpebralis superior lateralis*) of the superficial temporal artery (*A. temporalis superficialis*) (64). This distribution of blood vessels could confer a thermoregulatory advantage to water buffaloes by serving as a pathway for heat loss. Taylor (65) found that circulation around the horns allowed Toggenburg goats to serve as a pathway for thermoregulation. This could explain the increase in the temperature of the frontal-parietal region recorded in our study.

The thermal windows in the trunk regions, such as the abdominal or thoracic regions, had temperatures 2°C higher than those in the limbs region (*p* < 0.0001). This is related to the presence of vital metabolically active organs, such as the heart and liver, which contribute to raising body temperature as animals try to compensate through peripheral vasomotor changes in the structures of the limbs (66). Temperatures in these regions are produced by blood flow from the metatarsal dorsal and saphenous arteries (64). Changes in the limbs temperatures in dogs and horses have been associated with levels of muscle activity that increase blood flow at the local level (67, 68); however, the specific muscular response was not evaluated in our study.

The thermal windows of the nostrils were the one that presented the lowest temperatures of all nasal region (*Regio nasalis*) (*p* < 0.0001). Although this area has a high density of surface blood vessels from the maxillary artery and vein (24), elimination of water vapor during the respiratory cycle and tachypnea that occurs during stress are two factors that may influence nostril temperatures, leading to a greater loss of water vapor and heat (28). Finally, it is important to mention that a strong, positive, and significant correlation was observed among the thermal windows (*r* = 0.9, *p* < 0.0001). This means that as the temperature of one body region increases, those of other regions also increase because of the greater radiation of surface heat in the different thermal windows (69, 70).

In a similar manner, Napolitano et al. (71) the surface temperature of 109 buffalo calves was evaluated with the aim of recognizing variations in various thermal windows (both regions of the body and head) with respect to their birth weight, finding that the head region registers higher values (except the thermal window of the nostril) and with less variation with respect to pelvic limbs in all groups studied.

The results of the present study confirm the usefulness of the IRT technique for evaluating large ruminants, for example, to identify changes in the surface temperatures of buffaloes subjected to short periods of transport. However, it is important to emphasize that one of the limitations of this study is that we did not evaluate other response variables, such as physiological (e.g., body temperature) and endocrine parameters (e.g., cortisol concentrations), to relate the temperature increases detected to other factors. Likewise, another limitation of the present study could be the lack of monitoring the cleanliness of the animals to prevent that soiling, fecal matter, sweat, or urine could alter the thermal readings. The inclusion of physiological indicators during the pre- and post-transport phases could provide a clearer picture of the stress experienced by water buffaloes during transport. We consider this an important field for future explorations that will assess the degree of relationship between the surface temperatures of thermal windows and physiological indicators that are modified by the effects of transport.

## 5. Conclusion

According to the surface temperature values of water buffaloes transported for a short period, transport and the practices applied during this activity affect the temperature of various body and head regions. The responses of each region depend largely on the phase in which they are evaluated (from the paddock to post-transport). Herding and loading were identified as the moments when the buffaloes had the most marked stress responses, which increased the recorded thermal values. The strong positive correlations between the central and peripheral windows can help understand the importance of good handling of buffalo species during transport, with the goal of minimizing the potential stress they may experience and physiological responses that can impact their welfare.

## Data availability statement

The original contributions presented in the study are included in the article/supplementary material, further inquiries can be directed to the corresponding author.

## Ethics statement

Ethical review and approval was not required for the animal study because the animals used in this study were handled gently, without the use of physical utensils that could cause stress, in accordance with the guidelines of the Official Mexican Standards NOM-062-ZOO-1999, which establish technical specifications for the production, care, and ethical use of animals during experimentation; and NOM-051-ZOO which establish technical specifications for humanitarian handling in the mobilization of animals published by the Department of Agriculture, Rural Development, Fisheries and Alimentation. It is also important to consider that written informed consent was obtained from the owners for the participation of their animals in this study.

## Author contributions

All authors listed have made a substantial, direct, and intellectual contribution to the work and approved it for publication.

## Conflict of interest

The authors declare that the research was conducted in the absence of any commercial or financial relationships that could be construed as a potential conflict of interest.

## Publisher’s note

All claims expressed in this article are solely those of the authors and do not necessarily represent those of their affiliated organizations, or those of the publisher, the editors and the reviewers. Any product that may be evaluated in this article, or claim that may be made by its manufacturer, is not guaranteed or endorsed by the publisher.
